# Supplementary material for: Seasonal variation in water use for hygiene in Oromia, Ethiopia, and its implications for trachoma control: An intensive observational study
Source: PLoS Negl Trop Dis. 2022 May 13;16(5):e0010424. doi: 10.1371/journal.pntd.0010424 (PMC9173637; doi:10.1371/journal.pntd.0010424)
Supplement: Attachment — Submitted filename: PLOS NTDs Reviewer Response Letter.docx [file pntd.0010424.s001.docx]

Dear Reviewers,

Many thanks for your careful and thorough review of our manuscript (PNTD-D-21-01045) entitled Seasonal variation in water use for hygiene in Oromia, Ethiopia, and its implications for trachoma control: an intensive observational study.

Please find below a detailed response to your comments and queries. Edits to the manuscript and tables have been made with tracked changes for ease of review.

Kind regards,

Katie Greenland

| **Reviewer's Responses** | **Authors’ Response / Edits** |
| --- | --- |
| **Reviewer #1** | |
| Methods: There are several issues with the methods, as detailed in the comments. The sample size determination was not provided, the statistical analyses were not detailed sufficiently, and the population not described sufficiently. The most serious issues are around potential biases in these data, which are not discussed. | The reviewer has elaborated on these remarks with later comments – please see our responses to each point raised below. |
| Results: The results need clarification, especially Tables 3 and 4. Figure 1 can be deleted as described in the detailed comments. The analyses plan is not sufficiently detailed nor is the issue of clustering at household level addressed. | The reviewer has elaborated on these remarks with later comments – please see our responses to each point raised below.  We have decided to keep figure 1 and we believe it is useful for the reader to be able to visualise how life varies at the different times of year when data were collected. |
| Conclusions: There are several more limitations than the authors described, some with major impacts on the conclusions. Until the authors address concerns about the biases, it is not clear that some of the conclusions are valid | The reviewer has elaborated on these remarks with later comments – please see our responses to each point raised below. |
| Understanding water usage and washing practices in communities with trachoma is vital to designing intervention packages, and this manuscript provides data on a limited sample in Oromia, Ethiopia. These intense observational studies are difficult to do, and we do not expect the sample sizes typically seen in epidemiological studies. However, to be worthwhile for programs, as the authors state is the intent of the work, there must be some generalizability in a larger sense, and assurances that there are limited biases in the observations. These concerns are especially relevant when there are observations on just 9 households over just one day and a half in 3 different seasons. The authors must provide more information to the readers to assuage the concerns. Can the authors please provide the following: Selection of the sample 1. How was the sample size determined-both in number of sub-kebeles, number of households and number of days to represent the seasons/period? | This study was conducted immediately prior to a larger +200 household study in the same setting where we swabbed people and surfaces to look for *Chlamydia trachomatis* [(Last et al, 2020)](https://journals.plos.org/plosntds/article?id=10.1371/journal.pntd.0008120). We selected the kebeles to match the setting for the planned 200 household study, as we wanted to also use the first observation in the dry season to decide which surfaces to swab in the 200 household study. We have added to the Methods under the “Study Setting” to link to the subsequent study.  We selected 9 households pragmatically: we had 3 quali researchers and it was feasible for each field worker to observe 3 households in the time we had available for the study. We felt this was sufficient to enable us to observe patterns based on what we have seen in other studies. The number of days for observation was also based on feasibility as we had a range of different activities for our field team and just two drivers. We wanted to repeat the observation for reproducibility, but opted just to do this in the morning as that is a time when our previous work suggested that most hygiene-related washing takes place. We had already mentioned the small size of the study in our limitations. We have added remarks on the potential problems with the short length of the observation period (1 day and a morning in each season) now as well. |
| 2. What were the populations sizes of these sub-kebeles? | I’m afraid we don’t have this information anymore. |
| Line 83: It is said the sub-kebeles were “purposefully selected”. What were the criteria for selection? Out of how many sub-kebeles were these chosen? | See above (Point 1), selection of the kebeles for this study was determined by decisions on the location for the larger study and our clinical trial, and findings from this study fed into our larger research project. We have remarked on this in the methods. |
| Line 97. The households were also purposefully selected, but we only get a list of partial criteria, later in the manuscript: The houses had to have one preschool and one school age child, and one child had to have trachoma. We also learn that 21 children had trachoma-so how were the 9 households selected from among these households? Why did the households have to have trachoma? | We chose households with trachoma as we were interested to learn about behaviours that might be risk factors for trachoma. We have added more about the reason for the selection criteria in line 100 under subheading “Seasonal household observation”. |
| Bias: The authors have a very limited discussion of the potential for bias in these findings, but there are reasons to be concerned. Can the authors please provide information on the following:  It appears the trachoma survey was carried out prior to the observations, which means the families were aware of the status of their children. Given the emphasis on F ad E in the Ethiopian trachoma program, is it possible that families altered behavior for observers because they knew their child was affected? The authors need to discuss this in the manuscript and address this issue if they somehow do not feel it was a factor and why not. | We have revisited our discussion of bias in our limitations and strengthened it.  A convenience sample of children were checked for trachoma, but the families were not formally informed that their children had clinical signs of trachoma as all children were treated for trachoma. We did say that the study was linked to trachoma, but took care in the information we provided to avoid talking about trachoma prevention behaviours as we were very conscious not to induce reactivity. It is possible that some householders may have made the connection between trachoma and prevention behaviours such as face washing, and may have consequently modified their behaviour. However, most children washed their own faces and there was no evidence that adults were encouraging them to do this, or to use soap. If anything, we would expect that they would have known more about trachoma and why we were there on the second and third visit than on the first visit, yet the first visit in the dry season was when face washing and soap use was most common. We don’t believe this is therefore an important potential bias, but we do acknowledge it in the limitations section. |
| 6. Line 104. Were all the seasons observed in the same order for each household? That is, the first set of observations for all was dry season, then rainy season, then harvest season? If so, this is a serious limitation, as it is very possible that families increased washing practices and increased water availability for the first few days of observation, wishing to appear in good light to the observer, but then in subsequent seasons with more days, family practices settled down to normal routine. One way to address this is to have the first days of observation vary by season in different households and see if there is a temporal trend across seasons. Had different households started observations in different seasons one could parse out the effect of first observation from season.( Another way is to increase the number of days of observation in each season and determine if there is a change over the days; this was not done but was an option). As it is, the results show more water and more washing in the first set of observations which coincides with the dry season. The authors conclude there is more water usage and washing in dry season, rather than a Hawthorne effect of the timing of the initial observations. If there is bias, one would expect washing to decline over subsequent observations, which it does. The authors need a fuller discussion of this issue as this reviewer is unconvinced that there is more water in the household and more washing done in the dry season from these data. | Yes, all seasons were observed in the same order as all data were collected in 2018. This study led on to the development of an intervention and it wasn’t possible to allocate a longer period of time to the work to allow for observing different households in different orders.  We do not agree that washing practices would be likely to be subject to reactivity only on the first instance of observation. People were asked about washing practices through the survey *after* the dry season observation, so if anything, they would have been more aware of what we were doing and more likely to modify their behaviour accordingly during subsequent seasons, yet hygiene practices are not more frequent in these later rounds of data collection.  We have commented in the limitations on the length of the observation period in each season. |
| 7. The focus groups in these two sub kebeles were made aware of the study interest in water and washing. Were any of the households in the study part of the focus group? a. If so, this needs to be explicit. The discussion needs to reflect this major limitation , that washing as a focus of the research was known to some of the study participants. b. If not, how did the authors ensure that members of the focus group did not reveal the subject matter to the rest of the community. | The focus group discussions focussed on understanding a wide range of factors that varied seasonally, including water availability and rainfall. They did not discuss washing practices in these focus groups as we were conscious that people in the community may talk and our study members may have learnt more about what we were really interested in that we had said. None of the households included in the focus groups were part of the seasonal observation study. |
| 8. The authors state that the observers were unobtrusive, yet state that they observed nasal and ocular discharge not cleaned from the children’s faces after washing. Can the authors please address the following: a. How were the observers standardized? What was the definition used and what was the agreement between observers? b. It is not possible to ascertain ocular discharge without close observation. It is difficult to imagine how facial cleanliness was observed , right after washing a child’s face, unobtrusively. This would have indicated special interest in face washing in particular and again made participants aware of the study purpose. Can the authors please elaborate on this concern in the discussion and methods? | a) When we do clinical trials we standardise enumerators rigorously. For this type of study we did a week long training with didactic sessions and practical sessions each day. We paired enumerators in different pairings each day and checked they noticed and recorded the same things.  b) Observers were trained to be sure they did not immediately go over and inspect children’s faces after washing so as to avoid arousing suspicion that we were interested in face washing. This was something we were very aware of. They were taught to record visual cleanliness as soon as they could see the child’s face after washing. We have added some information on this to the methods and in the discussion we have noted that this was an imperfect assessment. |
| 9. Considering the above, how can the authors state the Hawthorne effect was not a problem (line 406), especially considering the temporal (rather than seasonal) changes discussed above | We have reframed and extended our discussion of the limitations of this study. |
| 10. Page 150: Significance testing is exceedingly difficult to do in these types of studies, because there are only 9 families, and data within each family are clearly correlated, as are observations over time in the same family/person. Longitudinal data analyses using repeated measures is reasonable, but some consideration must be given to clustering within families. It is difficult to see how a simple chi square could be used as it requires assumptions of independence and clustering within households is an issue. When comparisons with another population-based survey are used, how are the p values derived? Are they adjusted for household clustering? Please clarify how the p values were derived for testing within the 9-household survey. | We have reviewed the statistics with our statistician and have redone analysis where necessary to account for clustering of behaviour within households. We recalculated p values by fitting a random effects model instead of using chi square test. We have updated the p values where necessary and have altered the methods. Thank you for pointing this out. |
| 11. The manuscript has other areas that need more clarity for the reader. One is in the use of sessions and observation periods. In some places, there is a reference to 54 sessions (line 148) and others it is 27 sessions (line 229). In the methods, could the terms and numbers be clarified-did the authors mean that the nine households had six observation periods-one long first day, broken into two sessions plus two distinct periods for the harvest season? (Line 108)It is unclear. | The 54 sessions referred to in line 148 were 9 households, observed on two days (called two sessions) i.e. 18 sessions, in 3 seasons (18*3 = 54). In line 229 we spoke about half (14 of 27) of the day-long sessions, i.e. 9 households observed on one day in 3 seasons (9*3 = 27). The day one session in the harvest season was shorter as the enumerators did not stay overnight.  We appreciate that the reviewer does not find this clear and that other readers may experience the same and we have defined our use of “sessions” in the data analysis section where 54 sessions is first mentioned. |
| 12. This reviewer does not see the value of Figure one in the overall manuscript, or indeed the section on mapping. Besides clarifying the three observations periods, it is not really referenced or discussed further. This piece could be much reduced or deleted. | We found it immensely useful to understand how daily life varies at different times of year and would like to leave this in as we believe it could be useful for some readers who are not familiar with the creation of seasonal calendars. |
| 13. Table One; the 9 family households were more likely to have mobile phones, radios, and solar electricity. These markers of wealth, especially in rural Ethiopia, suggest the households chosen were different than the average households in this area. What is the impact on the findings from this difference-please add this to the discussion and consider the ramifications, especially in terms of generalizability. | Another reviewer also noted this. We have highlighted the difference in assets when we describe the findings in Table 1 and mentioned this in relation to generalisability in the discussion. |
| 14. In several places, the authors refer to how rare certain observations were (example-Laundry, line 260). Yet with only one day of observation , is it really so rare, or is the alleged scarcity a function of the few days observed. If laundry was done 2-3 times per week, which is rather a lot, it still is unlikely to be observed in the one observation day out of 7 days in a week. The authors need to temper their conclusions on the rarity of observations by acknowledging that the observation time itself was very limited. | We have conducted structured observation in a wide range of countries, and in most places clothes washing is observed almost daily, even during short observation sessions. This is notably different in this setting. We have run a lot of focus groups and done interviews and several unpublished observational studies elsewhere in Oromia and we consistently see and hear that laundry is done infrequently.  We agree with the reviewer that any activities occurring weekly as opposed to daily would be less likely to be observed in a study like this, and we have added notes on this in the discussion around line 394. |
| 14. Table 3 and 4 are exceedingly difficult to interpret. Are these numbers of times observed> Were the same number of persons observed at each season? what should we consider as a reference? How are we to interpret 112 instances of handwashing only in the dry season? Some benchmark is needed, like a per person or per day or something. The tables state these are column percentages, but they appear to be row percentages within seasons? | In response to comments from another reviewer, Table 3 has been altered to show data for a comparable time period in each season. The title header has been expanded to add clarity as well. The reviewer is correct, these are row percentages, we changed the table and hadn’t updated the footnote, so thank you for noticing this.    Interpreting 112 instances of handwashing in the dry season (now 80, as per changes to the table / text) should be based on comparing frequency of washing in general, and of soap use across the seasons.  Table 4 had an incorrect footnote as changes to the table had not been updated – this has also been corrected now. |
| 15. Please indicate the denominator for each person group, and the number not observed in each season. There is drop off over time, and this is not discussed. | In Table 4 we have added a column showing the denominator for each person group and a footnote to indicate the slight differences at harvest time due to 2 absences, one birth and one child moving up from pre-school to school-age group. |
| 15. line 230-233. Are we to understand that water collection was only observed on 13 of the 27 days of observation? And that there were 32 successful trips-on these 13 says? That is almost 3 trips per day, with none on the other days? How many families contributed to the 13 days and the 32 trips? It is just hard to understand how clustered these data are, and how limited by the few days observed. | That is all correct. Water was only collected 32 times in total on 13 of 27 day-long observations. All water collection was done by 6 households, 3 did not collect water on any observation day. This was unusual again, as in all other countries and settings, even when water was a 6 hour walk away, we have generally observed daily water collection, so we found this puzzling. |
| 16. in Table 2, there is almost a ten-liter difference, just in the dry season, between estimated water use and water use between 6:30 and 17:30 using the same method. Why was this so? Based on other statements, the authors feel most water use is in the morning, but in this case, it suggests virtually a third was used in the evenings. Does this mean then that for the dry season at least, the water consumption per person was a third higher, as it seems a third was used after 17:30? | I believe you are referring to estimated household water use calculated using Method 2, first during the whole observation period of 0630-2130 (38.0L) and secondly for the period of time that allows comparisons to be drawn across the seasons, from 0630-1730 (27.7L). Method 2 based on summing all observed instances of water use, is reassuringly accurate, as it is not very dissimilar to Method 1, based on water availability (42.6L). There is, as you note, almost 10L more water used after 1730 in the dry season, we don’t see this in the rainy season. Water use per capita is indeed higher in the dry season if you count the full observation period until 2130. It is also slightly higher in the rainy season, but not much. We have added a sentence to the text (line 274) to add these results. |
| 17. the authors state that water use for drinking was not collected because it was so modest. With between 5-8 persons per household, it is difficult to imagine that water use for drinking was not at least as great as that given to animals or to water the floor. Is the problem that water consumption could not be quantified for drinking? Or that water for drinking was often consumed off premises? Water for drinking and cooking are primary uses in my experience, admittedly in several other cultures, so it is difficult to reconcile the absence of this category in these data. | We have edited the wording in the methods so it is clear that we did not record water for drinking because people drank so little at any one time. It is true, over the course of the day in a household this could add up a bit, and this may partially explain the differences seen between Method 1 and Method 2 in our water consumption calculations. We have added a note in the discussion as a reminder that we did not track water use for drinking (around line 380). |
| Discussion The authors need to tone down their assumptions of the generalizability of this study and acknowledge its limitations. For example, in line 314, the implication is these data are representative of rural Oromia, which clearly cannot be the case. In line 230, the statement is made “and three of the nine households did not collect water from any source in any season”. Again, clearly not the case -the three households DID collect water at some point, just were not observed collecting water on the day of observation. Please go through and review the manuscript and temper such statements. | We have acknowledged more limitations in the discussion as per comments from the reviewers.  We have changed the wording in the first sentence of the discussion (previously line 314) in line with the reviewer’s comment.  Previous line 230 – that is very true, we have changed the wording.  We have checked the manuscript accordingly. |
| **Reviewer #2** | |
| Methods: Objectives of the study were well articulated and the researchers proposed an appropriate study design. Methods are adequately described. Although they included a low number of households for the study and the authors recognized that this might be a limitation, the findings of the study are important to contribute to understand better the challenges for trachoma elimination. The authors used adequate analysis methods to support the conclusions. Authors might review and consider to adjust the following in the Methods sections:  1. Line 84: TF stands for trachomatous inflammation-follicular not for follicular conjunctivitis. 2. Definition of "face washing practices" should be clarified for the purposes of the study. 3. Line 95: TI stands for Trachomatous inflammation– intense. | Points 1 to 3 have been addressed, with edits to the definitions of TF and TI and a clearer definition of how we have defined face washing. |
| Results are very well presented, clear, and matched the analysis plan. Please consider the following: 1. Line 169: TF stands for trachomatous inflammation-follicular not for follicular conjunctivitis. 2. Line 181: authors compared the characteristics of the 9 households studies to the wider population studied during the dry season in a survey in 2018. For this, authors listed the characteristics of both in table 1. It is not very clear that the characteristics are similar in both. For example, for characteristics such as radio ownership, mobile ownership, and having solar electricity, the 9 studied Households have much higher proportions than the population-based sample. Same happened with other characteristics (main water sources, water collection time, volume of water available in home, access to a latrine). It would be ideal if authors can explain a little bit why they arrived to the conclusion that there are no differences. 3. Line 391: it seems that authors missed to include the number of the reference. | 1. Corrected as suggested  2. The similarities we noted were related to education level, water sources, perceptions of water sufficiency, access to a latrine in use and soap availability. Given the small number of HHs in the study, the corresponding percentages change greatly with a change in 1 household, so we felt these were similar households with respect to WASH conditions. We have highlighted the difference in assets in the text and agree that there are some difference in markers of socio-economic status which we also note in the discussion.  3. Have deleted this part of the sentence. Thanks for noticing the lack of reference. |
| Conclusions are very well supported by the data presented by authors. Limitations were cleared described. Conclusions of this study are important to better understand challenges in making progress towards the elimination of trachoma as a public health problem. The international discussion about the need to better understand how to improve facial cleanliness can be enriched with the findings in this study. Understanding the practices in specific contexts, can help to tailor interventions to accelerate efforts toward the elimination goals. | Thank you. |
| Reviewer #2 This study explored a topic that is of high interest for the elimination of trachoma worldwide. There are very few studies involving the analysis of face washing practices in communities affected by trachoma that include direct observation of householders for several hours in different seasons of the year. This is a relevant study for countries affected by trachoma and to identify better interventions to tackle context-specific characteristics related to the endemicity of the disease. Congratulations to the authors for contributing with this study to the body of evidence on better practices for facial cleanliness. | Thank you. |
| **Reviewer #3** | |
| Study setting Page 5 line 84 – I would suggest including the name of the country and also to correct – follicular conjunctivitis” (TF) prevalence is high (TF1-9>40%) - “trachomatous inflammation – follicular (TF) prevalence is high (TF in children aged 1-9 years old >40%)” | Corrected as suggested. |
| Please correct throughout the manuscript “trachomatous inflammation – follicular (TF)”- Results page 9 line 169 and “trachomatous inflammation – intense (TI)”- page 5 line 96. | Corrected as suggested. |
| Data analysis Page 8 line 152- include one parenthesis at the beginning of the explanation Page8 line 155 – this is the first time that this symbol “∑ “appears – I suggest including an explanation how the sum of the amount of water was performed. | Thank you for noticing the missing parenthesis.  A brief explanation of how the sum of water was performed has been added. |
| Supplementary Table1 Many of the information already are described in the text. I think there is no need including the Supplementary Table1 in the manuscript. The information that is not ,can be included in the results section. | Have deleted supplementary table 1 as suggested. |
| Table 2 page 12 Please explain better the difference between “Estimated household water use during observation period” and “Estimated household water use for comparable period (06:30 - 17:30)”. I would suggest writing the information more clearly for better understanding “Estimated household water use during total observation period 06:30h-21:30h”. | Table 2 – have added further explanation so the differences are clearer, including a footnote. |
| Table 3 Lines 279 and 280 - it is written “ Data reported are for a comparable time period from 06:30 to 21:30 on Day 1 and from 0630 to 0930 on Day 2 for the dry and rainy seasons, and for a shorter observation session from 06:30h….” but at lines 271 to 274 says “Removing 272 washes that took place between 530pm and 930pm on Day 1 and before 6:30am on Day 2 from the 273 dataset removes 125 body washes, including 47 face washes (35 in the dry season (2 with soap), 274 in the rainy season (0 with soap) from the dataset”. Please explain the reason for removing the information of washes that took place between 17:30h and 21:30h from the analysis. There were removed from the table 3? | Table 3 reported all the data and hence was not comparable due to the shorter observation period during the harvest season. The text added in lines 271-274 was intended to explain this. However, we have decided to do this the other way round as agree that this could be clearer. Table 3 now reports only data that are from a comparable period. The text includes mention of the washes that this analysis excludes that were observed in the dry and rainy season. We hope you agree this is clearer and more informative. |
| Table 4 Include in the table that it is missing two information’s one father and one mother at the harvest time. | Have added this to the footnote. |
| Page 16 lines 289 and 290 – Please explain how these calculations were done. “Among those who washed theirs faces” (dry season – 55, plus rainy season 46, plus harvest time 37 = 138); dry season 55/138*100=40%; rainy season 46/138*100= 33%. My calculations do not result the number that it is written. | The text adds info not shown in the table as it is about frequency of washing during a comparable observation period in the dry vs rainy season.  Calculation as follows:  Dry season 55 individuals washed faces and 40 of these people washed their face more than once (40/55 =73% not 72%). Rainy season 46 individuals washed faces and 15 did so more than once (15/46 = 33%).  Have edited the text as follows:  *Among those who washed their faces* ***in each season****,* ***73%*** ***(40/55)*** *did so more than once in the dry season, compared with 33%* ***(15/46)*** *in the rainy season (p<0.001)* ***(data not shown).*** *NB: we recalculated the P values accounting for household clustering but this P value remained unchanged.* |
| Discussion Page 22 line 391 – “As part of wider activities to conserve water such as investing in innovative tools [ref]”…. I belie that the authors intended to include a reference there, but it is missing. | Have deleted this part of the sentence. Thanks for noticing this. |
| References Some references should be corrected, because are not Vancouver style. Please make necessary corrections of the references numbers 1, 4, 5 and 8. | Corrections have been made. |
| Keywords: Trachoma; water availability; hygiene, face washing; behaviour change. In the DECS/MeSH data base I did not find: water availability, face washing and behaviour change. There are: water; face and behaviour. I would suggest the DECS/MeSH Terminology – trachoma; prevention & control; health risk behaviours or risk reduction behavior, health promotion or health education. | Thank you, we have updated key words in line with suggestions. |
| General comments The manuscript presents a study conducted in Ethiopia exploring seasonal variation in hygiene behaviours to inform the development of face washing interventions for trachoma control. It is a very interesting paper that assessed hygiene habits in a community in the different seasons of the year. Shows how a different culture behave where water is scarce. It also presents suggestions for improving the F component of the SAFE strategy. I would suggest that the numbers of the percentages should be standardized, one number or without a number after the dot throughout the text.  At the abstract page 2 line 30, the hour is written “0630 and 2130” – the reader needs to guess that those numbers refer to hours. Only later in the text, that it was better explained. At the table 2 line 218 – it is written “06:30 am” and in page 14 line 272, it is written “530pm and 930pm”. Please refer to the hours and minutes and use the abbreviations units officially accepted for the use with the International System of Units (SI): Hours – h; minutes – min. Standardize the use throughout the text. I would suggest writing 06:30h or 1h48min. I would suggest including in all the tables’ title the country name and the date. | Thank you for your comments and review.  Percentages in the text are all standardised to a whole number, and percentages in tables to one decimal place. We have double-checked consistency.  Thank you for noticing discrepancies. |
