# Supplementary material for: Seasonal variation in water use for hygiene in Oromia, Ethiopia, and its implications for trachoma control: An intensive observational study
Source: PLoS Negl Trop Dis. 2022 May 13;16(5):e0010424. doi: 10.1371/journal.pntd.0010424 (PMC9173637; doi:10.1371/journal.pntd.0010424)
Supplement: Attachment — Submitted filename: Reviewer Response_PNTD-D-21-01045_R1_AL_Apr2022.docx [file pntd.0010424.s002.docx]

***Specific comments to reviewers are below in bold. I would again like to thank all reviewers for their detailed and insightful review of our manuscript.***

Reviewer #1: While I appreciate the responses to my concerns, there are still some major issues remaining. The request for sample size determination has not been addressed. The response of selecting 9 households pragmatically and issues of feasibility are certainly real issues but cannot be used to justify a study where measurement error is clearly a possibility. IF significance testing is important, as it appears to be in comparing across seasons, then some determination of sample size to detect differences must have been made, or at least some determination of the power to detect differences given the existing sample size. The generalizability issue is still problematic. The kebeles were selected to match another study and the houses were chosen in order of a convenience sample of children undergoing a survey and who had trachoma. It would seem prudent to severely limit the generalizability of these findings, especially in section staring line 435 which discusses implications for interventions. In the methods section, please provide the definitions used for ocular and nasal discharge, and some indicator of agreement among observers.

**Thank you for these additional comments. We have adjusted the limitations section to acknowledge the small sample size, particularly in the context of being underpowered for the statistical comparisons (Please see highlighted changes in Lines 481-484).**

**We have included the definitions for ocular and nasal discharge, which we have used in other studies (Please find highlighted changes in Lines 152-158). Observers were trained in the use of clear standard operating procedures to identify ocular and nasal discharge, but there was no formal assessment of inter-observer reliability, which we have noted in the manuscript (Please find highlighted changes in Lines 435-437).**

**We have also acknowledged the issues raised about generalizability beyond the region within which the study was conducted (Please find highlighted changes in Lines 459-465).**

Reviewer #1: The response to concern that the trachoma survey may have influenced the family’s behavior if they knew the status of their children was reassuring, and the manuscript should include a statement to the effect that the families were not informed that their children had signs of trachoma as all were treated regardless. I am still a bit unclear on the rationale for why, if families made the connection between trachoma and hygiene, increased face washing would be more apparent on the second or third visit than the first. In our experience with observations, it is in fact the first visit, where the families do not know the observer or what they are observing that they try and be on best behavior. The response provided was survey was done on reported water availability and use after observations in the dry and in the rainy season (line 164) but washing did not increase in the rainy and harvest seasons. While the survey may have changed behaviors in subsequent seasons, it does not address the concern that first observations could be biased due to the Hawthorne effect. In any case, the fact that the first observations were completely confounded by season is real, and possible bias could exist and needs to be refuted by data that this is not a source of bias on seasonal data than disagreement that it is not so. Or discussed as a possibility in limitations. Of note, the limitation section has been expanded but still does not address the Hawthorne effect of first observations.

**Thank you for these important comments. We have included a statement to acknowledge the fact that children and families were not informed of their trachoma status in this context at the time the study was conducted (Please see highlighted text Lines 118-122).**

**We have also acknowledged the potential for observer bias during the first season of observations, and to the best of our ability how we mitigated against that. We agree that this is a potential confounder, and hope that our changes to the manuscript have sufficiently acknowledged this (Please see highlighted text Lines 393-395 and 465-473).**

Reviewer #1: I remain unconvinced that there is real seasonality in the observations, given my concerns addressed above.

**We hope with the changes to the manuscript fully acknowledging the limitations with the study raised above that it remains of value for publication; we thank the reviewers and Editor for recognising that this is an important topic of public health relevance and of value when considering seasonal WASH interventions.**

Reviewer #3: There are still some hours that are not standardized. Please correct in lines 28, 30, 31, 197, 300 and 322.

**We would like to apologise for this oversight. These have all been corrected to a standardised format throughout the manuscript as requested.**
